# Comparison of the depth of vaccine-elicited HIV-1 Env epitope-specific CD8+ T lymphocyte responses

**DOI:** 10.1186/1742-4690-9-S2-P288

**Published:** 2012-09-13

**Authors:** SL Hulot, BT Korber, G Pantaleo, J Tartaglia, B Jacobs, B Perdiguero, CE Gomez, M Esteban, N Letvin, MS Seaman, B Haynes, S Santra

**Affiliations:** 1 Beth Israel Deaconess Medical Center, Boston, MA, USA; 2 Los Alamos National Laboratory, Los Alamos, NM, USA; 3 Centre Hospitalier Universitaire Vaudois (CHUV), Lausanne, Switzerland; 4 Sanofi Pasteur, Toronto, Canada; 5 Center for Infectious Diseases and Vaccinology, Tempe, AZ, USA; 6 Centro Nacional de Biotecnologia, Madrid, Spain; 7 Duke University Medical Center / Duke Human Vaccine Institute, Durham, NC, USA

## Background

One of the major challenges in the development of an effective HIV-1 vaccine is the extraordinary genetic diversity of the virus. Immunizations of nonhuman primates using consensus and mosaic immunogens have been shown to elicit cross-reactive CD8+T lymphocyte responses that increase the depth of epitope recognition. However, one of the limitations of vaccine-induced epitope-specific CD8+T lymphocytes includes lack of protection against diverse strains and emergent forms of HIV-1 due to altered T cell receptor (TCR) affinity for variant peptide:MHC class I complexes.

## Methods

In this study, we immunized a cohort of fifteen Mamu-A*01+ rhesus monkeys with either a 3-valent mosaic Env, group M consensus Env, or single clade B Env vaccine and compared the ability of the CD8+T lymphocyte populations elicited by each immunogen to recognize variants of an HIV-1 envelope epitope sequence p41A (YI9). We identified vaccine-induced CD8+T lymphocytes populations using tetramers constructed with 9 variants of p41A epitope. We assessed the ability of those variant peptides to activate CTL by measuring cytokine production and CD107a expression. We evaluated proliferation using carboxyfluorescein succinimidyl ester. Finally, we investigated the functional avidity of these CD8+T lymphocytes for the variant peptide:Mamu-A*01 complexes using surface plasmon resonance technology.

## Results

Our data show that Env immunizations can generate cross-reactive CD8+T lymphocytes that recognize 2 of 9 (22%) of the variants of p41A epitope, with higher responses induced by the consensus and the 3-valent mosaic immunogens (variant from clade C and variant from clade A/E) compared to the single clade Env immunogen. Tetramer-binding data also show that CD8+ T lymphocytes from monkeys immunized with mosaic immunogen have a trend of higher binding to majority of the variant peptides tested.

## Conclusion

This underscores the potential of mosaic immunogens for generating cellular immune responses with greater depth.

